# Time of Anderson-Fabry Disease Detection and Cardiovascular Presentation

**DOI:** 10.1155/2018/6131083

**Published:** 2018-03-20

**Authors:** K. Selthofer-Relatic

**Affiliations:** ^1^Department for Cardiovascular Disease, Osijek University Hospital, J. Huttlera 4, 31000 Osijek, Croatia; ^2^Department for Internal Medicine, Faculty of Medicine Osijek, Josip Juraj Strossmayer University of Osijek, Cara Hadrijana 10E, 31000 Osijek, Croatia

## Abstract

**Background:**

Anderson-Fabry disease is an X-linked inherited disease, which manifests in a different manner depending on gender and genotype. Making a working diagnosis of Anderson-Fabry disease is difficult because of several reasons: (a) that it is a multiorgan disease with wide variety of phenotypes, (b) different timelines of presentation, (c) gender differences, and (d) possible coexistence with other comorbidities. Late-onset/cardiac type of presentation with minimal involvement of other organs can additionally make diagnosis difficult.

**Aim:**

To describe different cardiac manifestations at different time points in the course of the disease: (1) 72-year-old female (echocardiography detection), heterozygote, significant left and mild right ventricular hypertrophy; (2) 62-year-old male (echocardiography detection), hemizygote, left ventricular hypertrophy, implanted cardiac pacemaker, a performed percutaneous coronary intervention after myocardial infarction, degenerative medium degree aortic valve stenosis; (3) 45-year-old female (asymptomatic/family screening), heterozygote, thickened mitral papillary muscle, mild left ventricular hypertrophy, first degree diastolic dysfunction; and (4) 75-year-old female (symptomatic/family screening), heterozygote, cardiomyopathy with reduced left ventricular ejection fraction after heart surgery (mitral valve annuloplasty and plastic repair of the tricuspid valve).

**Conclusion:**

All patients have Anderson-Fabry disease but with different clinical presentations depending on the gender, the type of mutation, and the time of detection. All these features can make the patients' profiles unique and delay the time of detection.

## 1. Introduction

Anderson-Fabry disease (AFD) is an X-linked disease of lysosomal metabolism resulting in attenuated activity or absence of the enzyme alpha-galactosidase A (a-Gal A). Impaired glycosphingolipid metabolism leads to systemic lysosomal globotriaosylceramide accumulation with multiorgan systemic involvement and complex clinical presentation: acroparesthesias, angiokeratoma, hypohidrosis, corneal and lenticular opacities, gastrointestinal and endocrine abnormalities, renal impairment, and neural and cardiovascular disease [1, 2].

AFD can affect both genders with 2 major phenotypes, the classic and the late-onset subtypes [3]. The classic phenotype has little or no a-Gal A activity and occurs with all typical AFD multiorganic presentations with renal failure development, hypertrophic cardiomyopathy, and cerebrovascular disease. Late-onset phenotype tends to have mutation-specific cardiac or kidney involvement, with slow progression and unknown underlying mechanisms [3]. Disease presentation of hemizygous males occurs more often as the classical type early in childhood or adolescence with typical symptoms, while heterozygous females can be affected as classical types, although the range of symptoms varies widely [2, 4]. So, it is more appropriate to describe AFD as a disease with a wide spectrum of heterogeneously progressive clinical phenotypes, from the classic severe phenotype in males to the asymptomatic disease course in females, with a variety of clinical presentations in-between. A high percentage of females develop vital organ involvement including the kidneys, heart, and/or brain about a decade later than the male patients [5, 6].

The typical cardiac AFD manifestation is concentric left ventricular hypertrophy (LVH) in up to 50% of males and one-third of females, with or without right ventricular hypertrophy (RVH), as a result of the globotriaosylceramide accumulation in cardiomyocytes, endothelial cells, smooth muscle cells, conduction tissue, and valvular fibroblasts with consequent complex cellular cascades and a final result of hypertrophy and fibrosis [2, 7]. ECG abnormalities, echocardiographic findings, and arrhythmias are part of clinical presentations depending on the disease stage. A typical cardiac symptom is angina as a result of microvascular disease, while myocardial infarction is uncommon [2, 8–10].

Everyday clinical practice in AFD detection is challenging because of several reasons: various clinical manifestations depending on gender, age, time of detection, and type of disease [11]. Disease severity variations (symptoms, ECG changes, echocardiographic signs, and presence of arrhythmias), together with other organ involvement, can lead to disease presentations with unique profiles, especially if we take into account alpha-galactosidase activity levels, biomarker lyso-GL-3 levels, and results of genetic testing. This case series presents 4 patients with different cardiovascular presentations (Table 1).

## 2. Case Presentation

### 2.1. Case 1

**Figure 1:**
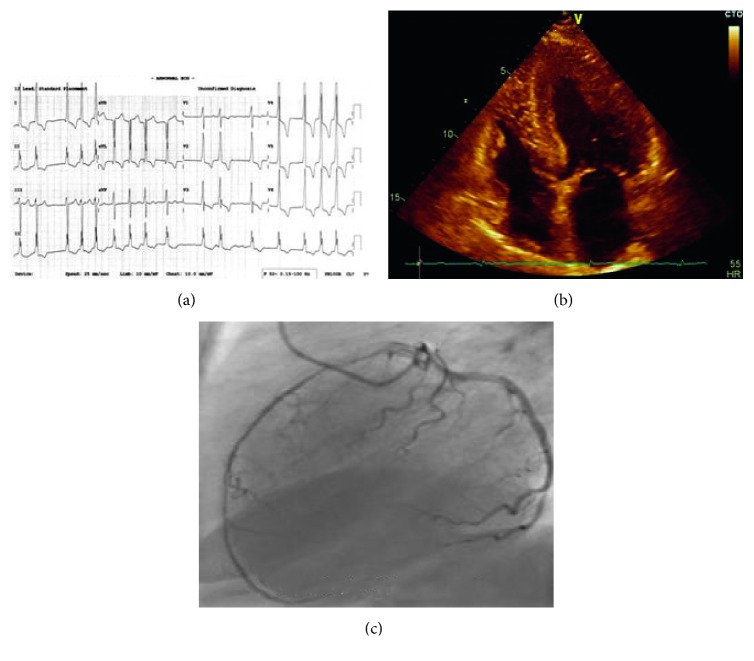
(a) Electrocardiogram: atrial fibrillation and left and right ventricular hypertrophy. (b) Two-dimensional transthoracic echocardiography (apical view): left and right ventricular hypertrophy. (c) Coronary angiography: normal findings of epicardial coronary artery.

A 72-year-old female presented with a personal history of hypohidrosis and acroparesthesia from childhood, started with antihypertensive medication, ramipril 1.25 mg, because of LVH and a mild level of hypertension 10 years ago. Also, laboratory analysis showed increased blood levels of creatinine (211 *µ*mol/L) and endogenous creatinine clearance (22.8 mL/min/1.73 m^2^) with proteinuria (174 mg/24 h). In 2013 and 2014, she had episodes of chest pain and dyspnea with increased levels of cardiac troponin and electrocardiogram changes (predominant left ventricular hypertrophy with negative T waves). On both occasions, she was admitted into the coronary unit with a working diagnosis of acute coronary syndrome. Coronary angiography was performed twice and showed no epicardial coronary artery stenosis, with developed smaller coronary arteries. During the past 2 years, she had several recidives of paroxysmal atrial fibrillation, started with oral anticoagulation. In last 10 years, echo examination was repeated several times, and every time significant LVH was described (LV mass calculated 305 g) while RVH was not. At the moment of detection, she had reduced ejection fraction of the left ventricle according to strain echo. Afterwards, testing for AFD was performed: alpha-galactosidase level was decreased (0.03 nmol/spot/21 h; normal range >0.185 nmol/spot/21 h) and a heterozygous mutation was detected (c.[758T>C]). Family screening test for sister and two sons were negative (Figures 1(a)–1(c)).

### 2.2. Case 2

**Figure 2:**
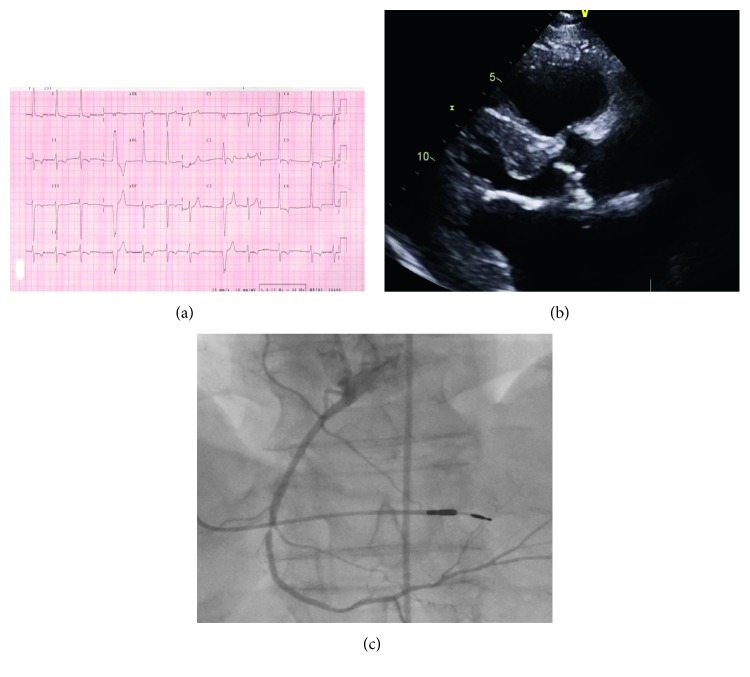
(a) Electrocardiogram: left ventricular hypertrophy and ventricular extrasystoles. (b) Two-dimensional transthoracic echocardiography (parasternal long-axis view): left ventricular hypertrophy and degenerative aortic valve changes. (c) Coronary angiography: significant right coronary artery stenosis and implanted cardiac electorstimulator.

A 62-year-old man presented with a personal history of hypohidrosis and acroparesthesia from childhood, hypertension over the last 20 years, started with cardiologic controls because of palpitations and dyspnea 20 years ago. At the moment of detection, he had persistent atrial fibrillation. He previously suffered an ischemic stroke, non-ST elevation myocardial infarction with percutaneous coronary intervention of the right coronary artery, had an implanted cardiac pacemaker because of sick sinus syndrome, increased blood levels of creatinine (116 *µ*mol/L) and endogenous creatinine clearance (78.6 mL/min/1.73 m^2^) with proteinuria (454 mg/24 h), without ophthalmological and dermatological manifestations of the disease. Repeated echo examination showed significant LVH without left ventricular outflow obstruction, decreased EF according to strain analysis, diastolic dysfunction of the first degree, and degenerative, moderate level of aortic valve stenosis. The patient was hospitalized for two more times because of chest pain with increased troponin levels. Afterwards, testing for Fabry disease was performed: alpha-galactosidase level was decreased (0.06 nmol/spot/21 h; normal range >0.12 nmol/spot/21 h), lyso-GL-3 was increased (36.1 ng/mL; normal range 0.0–3.5 ng/mL), and a hemizygous mutation was detected (c.[902G>T]) (Figures 2(a)–2(c)) [12].

### 2.3. Case 3

**Figure 3:**
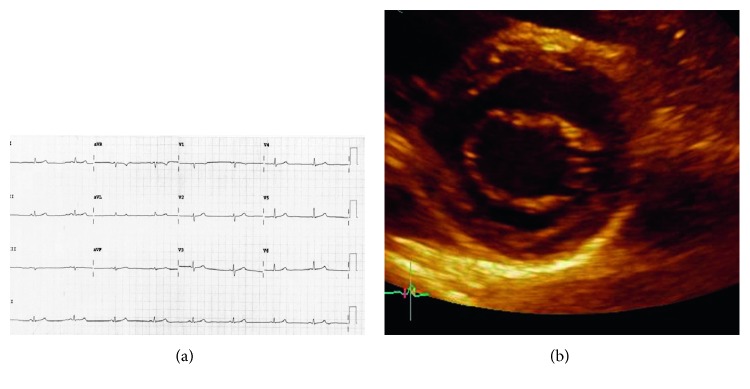
(a) Electrocardiogram: normal ECG. (b) Two-dimensional transthoracic echocardiography (short-axis view): normal mitral valve and partial thickened left ventricular walls.

An asymptomatic 45-year-old female was detected to be heterozygous during family screening (AFD affected father). Echocardiographic examination showed thickened mitral papillary muscle, LV wall thickness 11/12 mm, and mild degree of diastolic dysfunction. Testing for AF disease was performed: alpha-galactosidase level was normal (0.2 nmol/spot/21 h; normal range >0.12 nmol/spot/21 h) and a heterozygous mutation was detected (c.[902G>T]). Thereafter, heart MRI was performed; there was no late gadolinium enhancement or signs of myocardial fibrosis, examination considered as completely normal. Furthermore, brain MRI was normal. Renal biopsy showed typical zebra bodies located in podocytes and partly in epithelial tubular cells. Laboratory analysis showed normal renal function: creatinine level 76 *µ*mol/L, endogenous creatinine clearance 81 mL/min/1.73 m^2^, albuminuria 5.1 mg/L, and proteinuria 78 mg/24 h (Figures 3(a) and 3(b)).

### 2.4. Case 4

**Figure 4:**
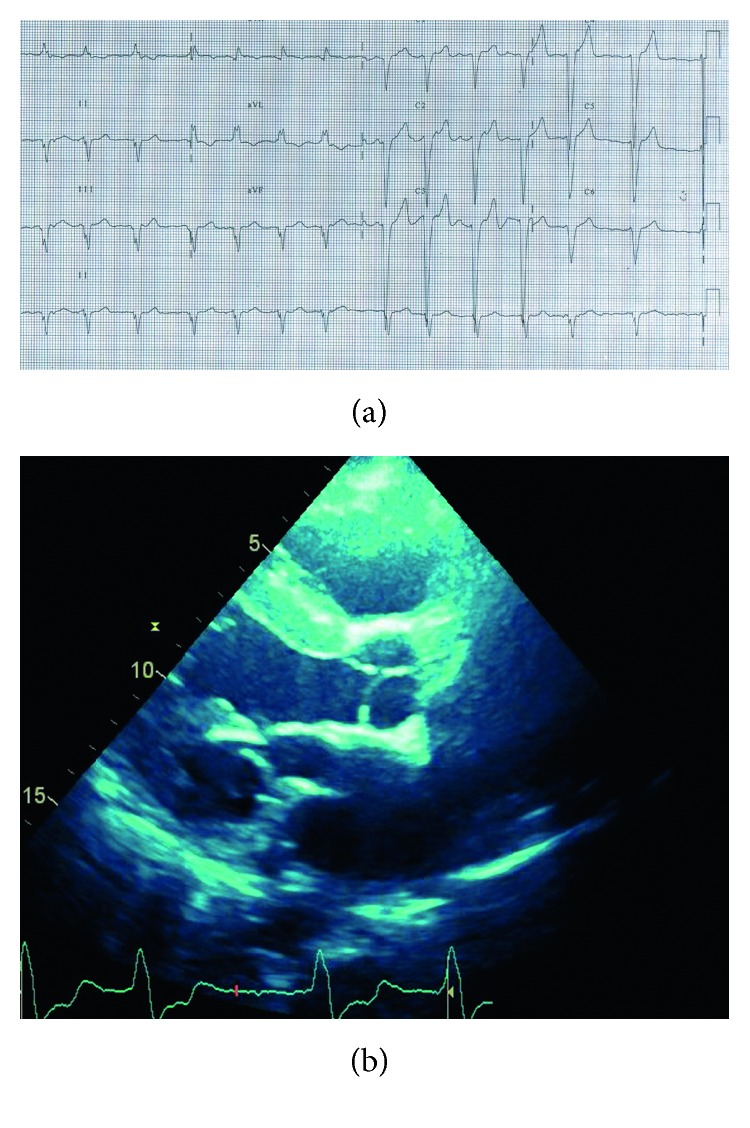
(a) Electrocardiogram: atrial fibrillation and left bundle branch block. (b) Two-dimensional echocardiography (parasternal long-axis view): hyperechogenic and hypertrophic interventricular septum; after mitral valve repair.

A 75-year-old female, heterozygote for AFD, detected in family screening, had hypertension history for over 20 years, atrial fibrillation, and dyspnea with signs of progressive heart failure of unknown cause during the past 10 years. Heart surgery was performed because of higher level of mitral and tricuspid regurgitation—mitral and tricuspid valve annuloplasty, before AFD detection. She had acroparestesia from childhood. According to earlier medical documentation, creatinine level was 125 *µ*mol/L, and proteinuria was described a bit over the reference values. The patient was not interested in further examinations. Echocardiographic examination was performed before testing: there were no typical signs of AFD, and she had moderate LVH with hyperechogenic/hypokinetic LV walls and reduced ejection fraction of left ventricle 30% according to two-dimensional transthoracic. Testing for Fabry disease was performed: alpha-galactosidase level was normal (1.7 *µ*mol/L/h; normal range >1.2 *µ*mol/L/h), lyso-GL-3 was increased (7.5 ng/mL; normal range 0.0–3.5 ng/mL), and a heterozygous mutation was detected (c.[902G>T]) (Figures 4(a) and 4(b)).

## 3. Discussion

Patients with AFD are usually detected during different times of their life and in different stages of the disease when typical hypertrophic cardiomyopathy is not always present (early or late stages).

The typical cardiac type of AFD presents with concentric or sometimes asymmetric LVH, with or without RVH. ECG changes include voltage signs of LVH/RVH, repolarization abnormalities, PR shortening, and different types of benign and malignant arrhythmias. Atherosclerotic coronary disease is not typical for AFD, but involvement of coronary microcirculation can lead to angina [2]. In the clinical routine, most patients are first evaluated with two-dimensional echocardiography. The echocardiographic determination is limited to morphology and function but without exact etiology determination. Cardiac magnetic resonance can evaluate the presence or absence of hyperenhancement and its location. Intramural or subepicardial areas of hyperenhancement, which are not related to any coronary perfusion territory, are typical for infiltrative disorders such as AFD. Additional valuable information can be gained by this examination, including information about the area of irreversible myocardial damage and prognosis, the amount of scarring, and the clinical risk for sudden death [13].

The hallmarks of AF cardiomyopathy (increasing wall thickness, regional functional abnormalities, and replacement fibrosis) during aging are not the same for both sexes. In male patients, the LV starts to hypertrophy during adolescence with concomitant reduction in longitudinal function. During aging, these two processes lead to replacement fibrosis. In females, the progression toward hypertrophy is prolonged, whereas the development of fibrosis and regional functional abnormalities progresses simultaneously [14]. Replacement fibrosis may be a valid screening tool in females as opposed to males in the early stages of AFD [15].

Symptoms and results of cardiac imaging may be different than expected: echocardiogram after cardiac surgery, coronary angiogram after percutaneous coronary intervention, or electrocardiogram after pacemaker implantation, and this can delay clinical assumptions and the decision for genetic testing.

A cardiologist is usually the first person who can raise suspicion about an AFD etiology in HCM with the help of transthoracic echocardiography. Knowing and thinking about HCM differential diagnoses is crucial. According to ESC HCM guidelines, in over 60% of adults with HCM, the disease is an autosomal dominant trait caused by mutations in cardiac sarcomere protein genes; in 5–10%, it is caused by other genetic disorders including inherited metabolic and neuromuscular diseases, chromosome abnormalities, and genetic syndromes; and about 25–30% are still of unknown cause [16]. In routine practice, the most common conditions causing LVH are arterial hypertension, aortic stenosis, and obesity [2].

In differential diagnosis, it is obligatory to expand our thoughts on several more facts about Anderson-Fabry disease: (1) gender difference (inheritance); (2) age difference (time of clinical presentation); (3) disease burden (depending on the affected organs and degrees of changes); (4) individual manifestation (unique pathophysiologic personal profiles); (5) presence of comorbidities (coexistence of different cardiovascular diseases).

## 4. Conclusion

From a cardiac point of view, patients with unexplained ventricular hypertrophy should be tested for AFD, even without other organ affection because of different genotypes and phenotypes of the disease. Patients with atypical echocardiographic LVH findings, with other organ affection, should also be tested for AFD, especially after invasive/operative cardiac therapeutic procedures.

## Figures and Tables

**Table 1:** Alpha-galactosidase levels and gene mutations of all cases.

	*α*-GAL level	Gene mutation
Case 1 (F)	**0.03** (normal range >0.185 nmol/spot/21 h)	c.[758T>C]
Case 2 (M)	**0.06** (normal range >0.185 nmol/spot/21 h)	c.[902G>T]
Case 3 (F)	0.2 (normal range >0.185 nmol/spot/21 h)	c.[902G>T]
Case 4 (F)	1.7 (normal range >1.2 *µ*mol/L/h)	c.[902G>T]
